# High expression of forkhead box protein C2 is associated with aggressive phenotypes and poor prognosis in clinical hepatocellular carcinoma

**DOI:** 10.1186/s12885-018-4503-6

**Published:** 2018-05-25

**Authors:** Yuki Shimoda, Yasunari Ubukata, Tadashi Handa, Takehiko Yokobori, Takayoshi Watanabe, Dolgormaa Gantumur, Kei Hagiwara, Takahiro Yamanaka, Mariko Tsukagoshi, Takamichi Igarashi, Akira Watanabe, Norio Kubo, Kenichiro Araki, Norifumi Harimoto, Ayaka Katayama, Toshiaki Hikino, Takaaki Sano, Kyoichi Ogata, Hiroyuki Kuwano, Ken Shirabe, Tetsunari Oyama

**Affiliations:** 1 Department of Diagnostic Pathology, Gunma University Graduate School of Medicine, 3-39-22 Showa-machi, Maebashi, Gunma 371-8511 Japan; 2 Department of General Surgical Science, Gunma University Graduate School of Medicine, 3-39-22 Showa-machi, Maebashi, Gunma 371-8511 Japan

**Keywords:** FOXC2, HCC, Epithelial-to-mesenchymal transition

## Abstract

**Background:**

Hepatocellular carcinoma (HCC) is one of the major causes of tumor death; thus, the identification of markers related to its diagnosis and prognosis is critical. Previous studies have revealed that epithelial-to-mesenchymal transition (EMT) is involved in tumor invasion and metastasis, and the forkhead box protein C2 (FOXC2) has been shown to promote tumor cell proliferation, invasion, and EMT. In the present study, we examined the clinicopathological significance of FOXC2 and EMT-related markers in clinical HCC specimens and identified factors related to the diagnosis and prognosis of HCC.

**Methods:**

The expression of FOXC2 and EMT-related markers was evaluated by immunohistochemistry in 84 cases of hepatocellular carcinoma.

**Results:**

A high expression of FOXC2 was observed in 26 of 84 cases, and expression was significantly correlated with background liver cirrhosis, poor tumor differentiation, high serum AFP, and elevated cell proliferation markers. In addition, this high expression was related to the induction of the Cadherin switch and vimentin expression and was an independent predictor for poor prognosis.

**Conclusion:**

The high expression of FOXC2 in HCC is correlated with tumor malignancy and poor prognosis, suggesting that FOXC2 may be an important prognostic factor for HCC.

## Background

Hepatocellular carcinoma (HCC) is a cancer with a poor prognosis [1]. Recently, advances in molecular target therapies towards advanced HCC have significantly improved the prognosis of patients with HCC [2]. However, patients with HCC with metastasis or refractory disease often require more effective and intensive therapeutic strategies. Therefore, to improve the prognosis of patients with HCC, novel therapeutic targets related to the malignant potential of HCC need to be identified.

Epithelial-to-mesenchymal transition (EMT) is an important cellular process which related to a developmental switch from an epithelial to a mesenchymal phenotypes [3, 4]. This process is essential for the embryonic development, moreover, EMT is also thought one of the vital molecular mechanisms inducing tumor invasion and metastasis. TGF-β is a pleiotropic factor that has a physiological function in regulating cell proliferation, differentiation, development, wound healing, and angiogenesis [5, 6]. In addition, TGF-β induces EMT, which has been well established as an important mechanism of cancer progression. Several down-stream transcription factors of TGF-β, such as the basic helix-loop-helix protein Twist, the zinc-finger proteins Snail and Slug, the E-box-binding protein ZEB1, and the forkhead box protein C2 (FOXC2), have been reported to induce EMT through the repression of E-cadherin expression, thereby playing pivotal roles in tumor metastasis [4, 7].

Functionally, FOXC2, also known as mesenchyme forkhead 1, is known as an important regulator of lymphangiogenesis [8], and FOXC2-knockout mice display a lymphedema-distichiasis syndrome [9]. FOXC2 was previously reported to function as an EMT-related gene during not only tumor progression but also organ repair [7, 10]. We previously reported the significance of FOXC2 expression in patients with esophageal cancer and cholangiocarcinoma, and a high expression of FOXC2 in tumor tissues was found to be related to cancer progression and a poor prognosis [11, 12]. These data were consistent with other cancers, including breast, colorectal, nasopharyngeal, esophageal, lung, ovarian, cholangiocarcinoma, and osteosarcoma [11–17]. Yang et al. have shown that the up-regulation of FOXC2 is associated with tumor size, vascular invasion, advanced TNM stage, promoting proliferation, and invasion in HCC [18]. However, few studies have examined the expressional relationship of FOXC2 and other EMT-related genes in HCC.

The purpose of this study was to clarify the clinical significance of FOXC2 and the relationship between FOXC2 and EMT-related proteins, such as E-cadherin, N-cadherin, and vimentin, in HCC. For this purpose, we carried out immunohistochemistry analysis to evaluate the relationships between FOXC2 expression, clinicopathological factors, and EMT-related proteins in clinical HCC samples.

## Methods

### Patient and samples

Eighty four patients with HCC who had undergone surgical resections at Gunma University Hospital between 1996 and 2014 were included in the study. The ages of the patients ranged from 48 to 89 years old. The tumor stage was classified according to the 6th Japanese tumor-node-metastasis (TNM) classification of Liver Cancer Study Group of Japan. Written informed consent for the collection of specimens was obtained from all participating patients with HCC, and the study protocol was approved by the local Ethics Committee.

### Tissue microarrays (TMAs)

Clinical formalin-fixed, paraffin-embedded (FFPE) samples were stored in the archives of the Clinical Department of Pathology, Gunma University Hospital. After reviewing the H&E-stained slides, two representative tumor area were marked on FFPE tissue blocks. These tumor areas were extracted as tissue core by using a cylinder. The diameter of tissue core was 2.0 mm. A manual arraying instrument (Beecher Instruments, Silver Spring, MD, USA) was used to assemble the paraffin blocks into TMAs, as previously described [19].

### Immunohistochemistry (IHC)

A 4-μm section was cut from paraffin blocks of samples. Each mounted sections were deparaffinized, rehydrated, and incubated with fresh 0.3% hydrogen peroxide in methanol for 30 min at room temperature to block endogenous peroxidase activity. The sections were then heated in boiled 10 mM citrate buffer (pH 6.0) at 98 °C for 30 min. Nonspecific binding sites were blocked by incubating with 0.25% Casein/1% BSA for 30 min at room temperature. Anti-FOXC2 primary monoclonal antibody (Abnova, Taipei, Taiwan) was used at a dilution of 1:100 at 4 °C overnight, as previously described [12]. The sections were washed in PBS, and the primary antibody was visualized using the Histofine Simple Stain MAX-PO (Multi) Kit (Nichirei, Tokyo, Japan) according to the instruction manual. The chromogen 3,3-diaminobenzidine tetrahydrochloride (Dojindo Laboratories, Kumamoto, Japan) was applied as a 0.02% solution containing 0.005% H_2_O_2_ in 50 mM Tris-HCl buffer (pH 7.6). The sections were lightly counterstained with Mayer’s haematoxylin and mounted. Negative controls were established by omitting the primary antibody. Other IHC was performed using the following primary anti-bodies: anti-E-cadherin (36; Ventana Medical Systems, Tucson, AZ, USA), anti-N-cadherin (6G11, Dako, Glostrup, Dermark), anti-Vimentin (V9, Dako), anti-ZEB1 (D91854, Atlas Antibodies, Stockholm, Sweden) and anti-Ki67 (30–9, Ventana).

### Immunohistochemical evaluation

The immunohistochemical FOXC2 expression was evaluated independently by two observers. A staining was primarily cytoplasmic in positive cases. The intensity of FOXC2 staining was scored as 0, 1+, 2+, and 3+. Grade 0, 1+, and 2+ staining was considered to be negative for FOXC2 expression, while grades 3 was considered to be positive. E-cadherin was evaluated by proportion score and intensity score. The proportion score are as follows: Score 0: < 10%; Score 1: 10–40%; and Score 2: > 40%. The intensity score are follows: Score 0: negative; Score 1: weak; Score 2: intermediate; and Score 3: strong. Finally, the two scores are combined, and a total score of 3 or more is regarded as positive. The expression of N-cadherin were defined as negative if membrane staining was detected in < 20% of tumor cells. Then we defined Cadherin switch as the samples showing both E-cadherin negative and N-cadherin positive. The expression of Vimentin in the cytoplasm of > 1% of tumor cells was defined as positive, regardless of staining intensity. The expression of ZEB1 in the nuclei of > 1% of tumor cells was defined as positive. The Ki67 labeling index was evaluated as the percentage of positive tumor cell nuclei based of > 500 tumor cells, regardless of staining intensity. The percentage > 1% was considered as high Ki67 labeling index. The IHC slides were evaluated by two independent pathologists in a blind manner.

### Statistical analysis

We used EZR (Saitama Medical Center Jichi Medical University; http://www.jichi.ac.jp/saitama-sct/SaitamaHP.files/statmed.html) for statistical analysis. The χ2 test and Fisher’s exact test were used to evaluate associations between clinicopathologic characteristics and FOXC2 expression intensities. To calculate and analyze overall survival and disease-free survival, we used the Kaplan–Meier method and the Log-rank test. Independent prognostic factors were tested by Univariate and multivariate analyses, which were based on the Cox proportional hazards regression model. A statistically significant *p*-value was considered to be less than 0.05.

## Results

### Immunohistochemical staining of FOXC2 in HCC tissues

**Fig. 1:**
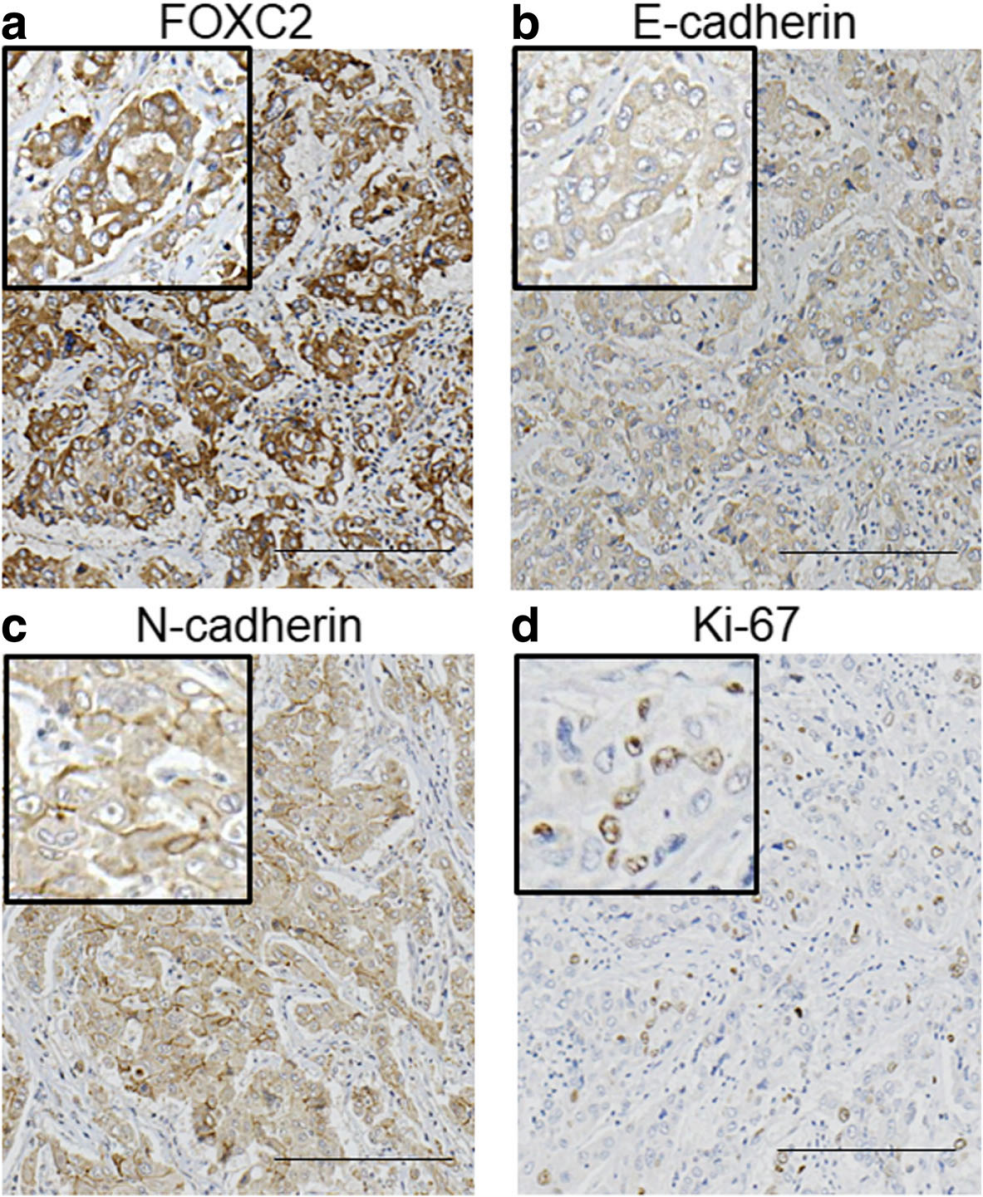
Immunohistochemical analysis of FOXC2, E-cadherin, N-cadherin, and Ki-67 in representative HCC tissues from an identical patient. **a** High FOXC2 expression in an HCC tissue; **b** Low E-cadherin expression in an HCC tissue; **c** High N-cadherin expression in an HCC tissue; **d** High Ki-67 expression in an HCC tissue. Scale bar, 200 μm

The expression of FOXC2 was evaluated in 84 HCC samples by immunohistochemistry. Cytoplasmic staining of FOXC2 was primarily observed (Fig. 1a). In total, 26 of 84 HCC samples (31%) were considered high for FOXC2 expression, whereas 58 HCC samples (69%) were considered low for FOXC2 expression.

### Expression of FOXC2 in HCC tissues and its correlation with clinicopathological factors

Correlations between FOXC2 expression in HCC samples and patient age, gender, liver cirrhosis, T classification, differentiation, tumor size, pattern of tumor growth, Fc-inf, IM, Vv, Vp, Va, serum AFP level, serum PIVKAII level, and the Ki-67 labeling index are shown in Table 1. High FOXC2 expression was correlated with liver cirrhosis (*P* = 0.0296), poor differentiation (*P* = 0.0302), high serum AFP levels (*P* = 0.00078), and the Ki-67 labeling index (*P* = 0.0348).

**Table 1:** Clinicopathological characteristics of patients with hepatocellular carcinoma (HCC) according to forkhead box protein C2 (FOXC2) expression

Parameters	FOXC2		*P* value
	Low expression*n* = 58 (%)	High expression*n* = 26 (%)	
Age (years)			
< 65	18 (31.0)	6 (23.1)	0.603
≥ 65	40 (69.0)	20 (76.9)	
Gender			
Male	46 (79.3)	19 (73.1)	0.578
Female	12 (20.7)	7 (26.9)	
Liver cirrhosis			
Negative	40 (69.0)	11 (42.3)	0.0296^a^
Positive	18 (31.0)	15 (57.7)	
T classification			
1	5 (8.6)	3 (11.6)	0.333
2	20 (34.5)	5 (19.2)	
3	28 (48.3)	13 (50.0)	
4	5 (8.6)	5 (19.2)	
Differentiation			
Well or Moderate	57 (98.3)	22 (84.7)	0.0302^a^
Poor	1 (1.7)	4 (15.3)	
Tumor size (mm)			
≤ 20	5 (8.6)	4 (18.2)	0.449
> 20	53 (91.4)	22 (81.8)	
Pattern of tumor growth			
Eg	49 (84.5)	18 (55.6)	0.143
Ig	9 (15.5)	8 (44.4)	
Fc-Inf			
Negative	37 (63.8)	11 (42.3)	0.0948
Positive	21 (36.2)	15 (57.7)	
IM			
Negative	50 (86.2)	20 (76.9)	0.347
Positive	8 (13.8)	6 (23.1)	
Vv			
Negative	46 (79.3)	22 (81.8)	0.766
Positive	12 (20.7)	4 (18.2)	
Vp			
Negative	38 (65.5)	17 (65.4)	1
Positive	20 (34.5)	9 (34.6)	
Va			
Negative	55 (94.8)	25 (96.2)	1
Positive	3 (5.2)	1 (3.8)	
AFP level (ng/ml) (*n* = 73)			
Normal (≤10)	27 (55.1)	3 (12.5)	0.000777^a^
High (> 10)	22 (44.9)	21 (87.5)	
PIVKA II level (AU/ml) (*n* = 69)			
Normal (≤40)	19 (39.6)	11 (52.4)	0.43
High (> 40)	29 (60.4)	10 (47.6)	
Ki67 labeling index			
< 1%	21 (36.2)	3 (11.5)	0.0348^a^
≥ 1%	37 (63.8)	23 (88.5)	

^a^Statistical significance is indicated by *P* < 0.05. Eg (expansive growth); Boundary between cancer and surrounding liver tissue is clear. Ig (infiltrative growth); Boundary between cancer and surrounding liver tissue is unclear

### Relationship between FOXC2 expression and EMT-related markers

We examined the relationship between FOXC2 expression and IHC staining of the EMT-related markers E-cadherin, N-cadherin, vimentin, and ZEB1. High FOXC2 expression had a strong association with the induction of the Cadherin switch and a high expression of vimentin (Table 2; *P* = 0.0414 and *P* = 0.0273) (Fig. 1).

**Table 2:** The relationship of FOXC2 expression and EMT-related markers in 84 HCC samples

Parameters	FOXC2		*P* value
	Low expression *n* = 58 (%)	High expression *n* = 26 (%)	
E-cadherin			
Negative	23 (39.7)	13 (50)	0.476
Positive	35 (60.3)	13 (50)	
N-cadherin			
Negative	31 (53.4)	16 (61.5)	0.635
Positive	27 (46.6)	10 (38.5)	
Cadherin switch			
Negative	53 (91.4)	19 (73.1)	0.0414^a^
Positive	5 (8.6)	7 (26.9)	
Vimentin			
Negative	58 (100)	23 (88.5)	0.0273^a^
Positive	0 (0)	3 (11.5)	
ZEB1			
Negative	32 (55.2)	16 (61.3)	0.639
Positive	26 (44.8)	10 (38.7)	

^a^Statistical significance is indicated by *P* < 0.05

### Prognostic significance of FOXC2 expression in HCC

**Fig. 2:**
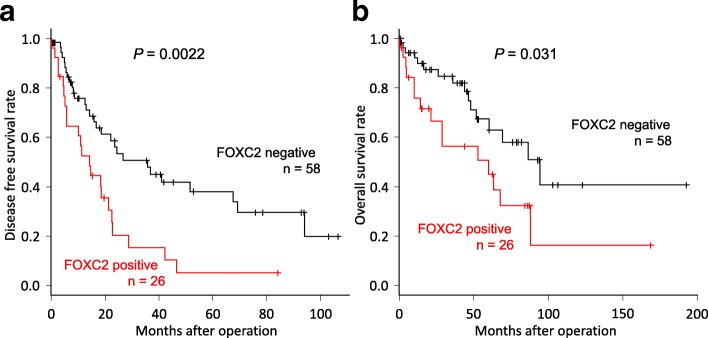
Relationship between postoperative survival and FOXC2 expression in 84 patients with HCC. Kaplan–Meier curves of the low expression of FOXC2 and high expression of FOXC2 groups are shown. **a** A high expression of FOXC2 indicated a poor prognosis for the disease-free survival rate (*P* = 0.0022). **b** A high expression of FOXC2 indicated a poor prognosis for the overall survival rate (*P* = 0.031)

Disease-free survival rates (DFS) and the overall survival rates (OS) of patients with HCC are shown in Fig. 2. Patients with high FOXC2 expression had significantly poorer prognoses than patients with low expression in both DFS (*P* = 0.0022) and OS (*P* = 0.031). For OS, FOXC2 expression was a prognostic factor for poor survival in a univariate analysis (Table 3; RR 2.14, 95% CI 1.05–4.34, *P* = 0.035). Additionally, the pattern of tumor growth, T classification, and portal vein invasion were also prognostic factors in the univariate analysis. In the multivariate analysis, FOXC2 expression was an independent prognostic factor for poor survival (Table 3; RR 2.21, 95% CI 1.06–4.57, *P* = 0.033).

**Table 3:** Results of univariate and multivariate analyses of clinicopathological factors affecting the overall survival rate following surgery

Clinicopathologic variable	Univariate analysis			Multivariate analysis		
	RR	95%CI	*P* value	RR	95%CI	*P* value
FOXC2 expression (low/high)	2.14	1.05–4.34	0.035^a^	2.21	1.06–4.57	0.033^a^
Age (≤65/> 65)	1.17	0.518–2.66	0.70	–	–	–
Gender (male/female)	1.13	0.485–2.62	0.78	–	–	–
Liver cirrhosis (negative/positive)	1.86	0.913–3.77	0.087	–	–	–
Differentiation (well or moderate/poor)	1.30	0.307–5.49	0.72	–	–	–
Pattern of tumor growth (Eg/Ig)	3.29	1.53–7.07	0.0023^a^	3.06	1.37–6.80	0.0062^a^
T classification (T1–3/T4)	4.33	1.82–10.34	< 0.001^a^	3.82	1.18–12.37	0.025^a^
Portal vein invasion (negative/positive)	2.19	1.03–4.65	0.042^a^	1.31	0.477–3.59	0.6
AFP level (normal/high)	1.24	0.529–2.91	0.62	–	–	–
PIVKA II level (normal/high)	0.749	0.323–1.73	0.50	–	–	–

*RR* relative risk, *CI* confidence interval

^a^Statistical significance is indicated by *P* < 0.05

## Discussion

In the present study, we demonstrated that a high expression of the EMT inducer, FOXC2, in primary HCC samples is associated with liver cirrhosis, malignant potential, high serum AFP, and poor prognosis. Moreover, FOXC2 accumulation was related to the induction of the Cadherin switch and increased expression of the mesenchymal marker vimentin.

Yang et al. have indicated that high expression of FOXC2 is related to tumor size, vascular invasion, advanced TNM stage, and promoting proliferation and invasion in HCC [18]. They focused on AKT-mediated MMP-2 and MMP-9 to explain FOXC2-related cancer aggressiveness. Conversely, by focusing on a correlation between high FOXC2 and Cadherin switch, we showed that high expression of FOXC2 in clinical HCC samples is involved in EMT-related tumor aggressiveness.

High expression levels of FOXC2 in HCC were significantly associated with the low expression of E-cadherin/high N-cadherin, termed the Cadherin switch, and accumulation of the mesenchymal marker vimentin. FOXC2 has been reported to be an important EMT inducer via TGF-β signaling in several cancers [7, 17, 20, 21]. Moreover, FOXC2 can induce the expression of cancer-related genes, including AKT, GSK3β, and Snail [22]. The activation of the AKT-GSK3β-Snail signaling pathway in colon cancer has been previously reported to induce EMT. From these observations, it is suggested that suppression by targeting FOXC2 may be important to overcome EMT in patients with clinical HCC.

High expression of FOXC2 in HCC was significantly associated with the progression of cirrhosis in the background liver. Hepatic cirrhosis is known to be induced by viral infection, alcohol, fatty liver, and non-alcoholic steatohepatitis [23, 24]. At that time, it was reported that the activation of the TGF-β/Smad signal is likely an important key regulator, and TGF-β inhibitors suppress hepatic fibrosis [25]. In breast cancer cell lines, FOXC 2 is known to be induced by the activation of TGF-β signaling [7]. These data suggest that TGF-β signaling may be one of the main FOXC2 regulation mechanisms and that hepatic cirrhosis, induced by TGF-β signaling, may cause HCC expressing high FOXC2 with aggressive phenotypes.

In the present study, we clarified the positive correlation between FOXC2 expression and Ki-67 accumulation. Cui et al. previously reported that FOXC2 can facilitate the proliferation ability in pancreatic cancer via the activation of β-catenin/TCF signaling, which is well known as a proliferation regulator in cancer cells [26]. Moreover, FOXC2 has been known to promote cell proliferation through the activation of MAPK and AKT pathways [27]. Moreover, we previously reported that FOXC2 suppression by RNA interference induces anti-proliferative activity in esophageal cancer cells and cholangiocarcinoma cells [11, 12]. Thus, FOXC2 may be an important regulator in cancer proliferation in not only HCC but also other cancers.

Many studies have reported that FOXC2 is related to chemotherapeutic resistance in several cancers [28–31]. Indeed, FOXC2 suppression may inhibit EMT induction and multidrug resistance in basal-like breast cancer and nasopharyngeal cancer [30, 32]. On the other hand, Zang et al. clarified that FOXC2 accumulation, induced by long non-coding RNA FOXC2-AS1, can increase the expression of the multidrug-related gene ATP binding cassette subfamily B member 1 (ABCB1) [33]. As mentioned above, FOXC2 appears to be related to cancer aggressiveness, including proliferative marker accumulation and EMT induction/Cadherin switch in HCC. Targeting FOXC2 may be effective to overcome aggressive phenotypes and therapeutic resistance in HCC.

Interestingly, Yu et al. reported a new FOXC2-targeting strategy using the natural compound resveratrol, which is known as a beneficial compound found in red wine, which suppressed FOXC2 expression in lung cancer cells via miR-520 h suppression [34]. Actually, resveratrol has been reported to suppress the viability of HCC cells [35–37]; therefore, the administration of resveratrol in patients with clinical HCC may be a good therapeutic candidate to overcome HCC aggressiveness via targeting FOXC2.

## Conclusions

In conclusion, we have shown that FOXC2 expression in HCC is associated with several factors, including poor survival, poor differentiation, serum AFP levels, proliferation marker Ki67 expression, and the Cadherin switch. High FOXC2 expression levels may be a powerful marker of aggressive phenotypes and poor survival in patients with HCC.

## Abbreviations

ABCB1: ATP binding cassette subfamily B member 1
DFS: Disease-free survival rates
EMT: Epithelial-to-mesenchymal transition
FFPE: Formalin-fixed, paraffin-embedded
FOXC2: Forkhead box protein C2
HCC: Hepatocellular carcinoma
IHC: Immunohistochemistry
OS: Overall survival rates
TMAs: Tissue microarrays
TNM: Tumor-node-metastasis
